# Retraction: A study of the economic growth effects of market integration: An examination of 27 cities in the Yangtze River Delta city cluster

**DOI:** 10.1371/journal.pone.0349818

**Published:** 2026-05-28

**Authors:** The PLOS One Editors

The *PLOS One* Editors retract this article [1] due to concerns about potential manipulation of the publication process. These concerns call into question the validity and provenance of the reported results. We regret that the issues were not identified prior to the article’s publication.

The author either did not respond directly or could not be reached.

## References

[1] Yang J. RETRACTED: A study of the economic growth effects of market integration: An examination of 27 cities in the Yangtze River Delta city cluster. PLoS One. 2023;18(11):e0287970. doi: 10.1371/journal.pone.0287970 38033003 PMC10688701

